# *Ocimum basilicum* (Basil) Modulates Apoptosis and Neurogenesis in Olfactory Pulp of Mice Exposed to Chronic Unpredictable Mild Stress

**DOI:** 10.3389/fpsyt.2020.569711

**Published:** 2020-09-11

**Authors:** Nasra N. Ayuob, Maha J. Balgoon, Soad Ali, Ibrahim S. Alnoury, Hailah M. ALmohaimeed, Amany A. AbdElfattah

**Affiliations:** ^1^Department of Medical Histology and Cell Biology, Faculty of Medicine, Damietta University, Damietta, Egypt; ^2^Department of Medical Histology, Faculty of Medicine, Delta University for Science and Technology, Mansoura, Egypt; ^3^Yousef Abdullatif Jameel, Chair of Prophetic Medical Applications (YAJCPMA), Faculty of Medicine, King Abdulaziz University, Jeddah, Saudi Arabia; ^4^Department of Biochemistry, Faculty of Science, King Abdulaziz University, Jeddah, Saudi Arabia; ^5^Department of Anatomy, Faculty of Medicine, King Abdulaziz University, Jeddah, Saudi Arabia; ^6^Department of Histology, Faculty of Medicine, Assuit University, Assuit, Egypt; ^7^Department of ENT, H&N Surgery, Faculty of Medicine, King Abdulaziz University Hospital, Jeddah, Saudi Arabia; ^8^Department of Basic Science, Medical College, Princess Noruh bint Abdulrahman University (PNU), Riyadh, Saudi Arabia; ^9^Department of Histology and Cell Biology, Faculty of Medicine, Mansoura University, Mansoura, Egypt

**Keywords:** *Ocimum basilicum*, chronic stress, caspase-3, anti-glial fibrillary acidic protein, olfactory bulb, neurogenesis

## Abstract

**Background:**

*Ocimum basilicum* *(O. basilicum*) was described to have antidepressant and anxiolytic activities. Although the relationship between the main olfactory bulb (MOB) and depression was recently reported, the chronic stress-induced dysfunction of the MOB is not clearly described.

**Objectives:**

This study aimed to assess the efficacy of inhalation of *O. basilicum* essential oils in improving chronic unpredictable mild stress (CUMS)-induced changes in MOB of mice and understand the mechanism underlying such effect.

**Materials and Methods:**

Adult male mice (n=40) were assigned into four groups included the control, CUMS-exposed, CUMS + fluoxetine (FLU), CUMS + *O. basilicum*. Behavioral changes, serum corticosterone level, and gene expression of GFAP, Ki 67, and caspase-3 were assessed using real-time PCR (RT-PCR). Histopathological and immunochemical examination of the MOB was performed.

**Results:**

FLU and *O. basilicum* significantly down-regulated (p = 0.002, p<0.001) caspase-3 gene expression indicating reduced apoptosis and up-regulated (p = 0.002, p < 0.001) Ki67 gene expression indicating enhanced neurogenesis in MOB, respectively. FLU and *O. basilicum*-treated mice markedly improved MOB mitral cell layer distortion and shrinkage induced by CUMS.

**Conclusion:**

*O. basilicum* relieved both biochemically and histopathological chronic stress-induced changes in the main olfactory bulb possibly through up-regulation of gene expression of GFAP and Ki67 and down-regulation of caspase-3 in the MOB.

## Introduction 

Chronic stress results in deterioration in mood, cognition, memory and may be a leading cause in the occurrence of many systemic diseases as Parkinson’s disease, type 2 diabetes, gastric ulceration, and Alzheimer’s disease (1).

Olfactory system is distinctive as it is considered the most proliferative CNS system entertaining differentiating progenitor cells, migrating from the subventricular zone to the olfactory bulb (OB), where they differentiate into tyrosine hydroxylase (THC) or GABA (C) interneurons (2). The main olfactory bulb (MOB) is an important part of the olfactory system that results in post developmental neurogenesis (3).

Unpredictable stress is associated with atrophy in cortical and limbic brain regions including the MOB. It was reported that olfactory deficits often accompany neurodegenerative diseases. This was noticed in idiopathic Parkinson’s disease where advanced olfactory deficits were initial signs of the disease that occurs before the first motor dysfunctions (4). However, the mechanism of the dysfunction & the structural changes of MOB resulted from CUMS is still mostly unidentified (3). The model of (CUMS) is one of the common and verified models used to study depression. This model is characterized by the occurrence of long-lasting neurodegenerative changes including behavioral, neurochemical, and neuroendocrinal changes that mimic those observed in depressed patients (5).

Aromatherapy is the utilization of aromatic essences and pure essential oils, naturally-extracted from roots, leaves, and flowers of plants in promoting the health of body, mind as well as in treatment of some diseases. It was reported that it is potentially useful in the management of disruptive behaviors and the reduction of agitation in people with dementia (6, 7). *Ocimum basilicum* (*O. basilicum*), also called basil or Raihan, is an annual plant used as a spice for culinary purposes in salads and pasta sauces. It is extensively utilized as a flavoring agent and in production of perfumes and soap (8, 9). In addition, it is used for medicinal purposes due to its antidepressant, anxiolytic, sedative activities (10, 11). *O. basilicum* as a therapeutic supplement has been investigated in many animal models of cognitive deficits (12, 13). Recently, the therapeutic efficacy of *O. basilicum* against impairment of memory function in animal model of Multiple sclerosis was reported (14).

Although the relationship between MOB and depression was recently reported (15–17), the CUMS-induced dysfunction of MOB is not clearly described. Therefore, the current research was designed to evaluate the CUMS-induced alterations occurred in MOB in mice exposed to mild chronic depression and the efficacy of inhalation of *O. basilicum* essential oils in improving these changes as well as understand the possible mechanism underlying this effect.

## Material and Study Design

Ethical approval and guidelines of animal care were obtained from “the biomedical research ethics committee” and King Fahad Medical Research Center (KFMRC), King Abdulaziz University (KAU), Jeddah, Saudi Arabia (SA).

Male Swiss albino mice (N=40), aged 5 weeks, and weighed (30–40 g), were obtained from the KFMRC and were maintained for 2 weeks before the experiment at 27 ± 1°C, were fed on a standard mice pellets and water *ad-libitum* in order to acclimatized and ensure normal growth and behavior.

### Chemicals

Fluoxetine (FLU) is a selective serotonin reuptake inhibitor used as antidepressant to treat CUMS-induced depression in mice of the positive control group. It was purchased from Dar Al Dawa (DAD) Pharmaceuticals Co., Ltd. (Amman, Jordan). Sodium carboxymethyl cellulose (CMC-Na, 0.03%) was used to dissolve FLU and 20 mg/kg was given to the mice by intragastric gavage (18). *O. basilicum* was collected from the Jeddah gardens. A botanist from the Faculty of Science, KAU helped to morphologically identify *O. basilicum*. The essential oil of *O. basilicum* was prepared according to the method of (19). The constituents of *O. basilicum* essential oil were identified using “gas chromatography coupled to mass spectrometry” (GC-MS; Agilent, Columbia, MD). Essential oils of *O. basilicum* were diluted first with 5% propylene glycol before their use (Sigma, St. Louis, MO) as reported by (20), then given through inhalation. Amyl acetate, 5% (Sigma), was given to untreated CUMS mice by inhalation as it was reported by (21) that it has no effect on anxiety.

### Experimental Design

After acclimatization period, the mice were assigned into four groups (n=10 each) at random. Each five mice were held together in a cage. The four groups included the control, CUMS, CUMS + FLU, CUMS + *O. basilicum*. The CUMS procedure used in this study was previously described. Mice were exposed to CUMS for continuous 4 weeks followed by 2 weeks treatment by amyl acetate, FLU, or *O. basilicum*.

Inhalation of *O. basilicum* and amyl acetate was performed according to Chioca, Ferro (20) using a “32 × 24 × 32 cm odor-isolated acrylic box”.

### Behavioral Assessment

Behavior tests were done after 6 weeks between 8:00 and 11:30 AM [Mineur, Belzung (22)] using the elevated plus maze test (EPM) and the forced swimming test (FST) spaced by a 24 h between tests.

The FST was carried as described by Doron, Lotan (23). The total time spent not moving by the mouse during 6 min was recorded in seconds as previously described by Ayuob, Firgany (24). Regarding the EPM, its procedure was described by Ali, Abd El Wahab (10). The number of times the mouse enter to the closed arm during 6 min and the time spent by each mouse inside the open and closed arms were registered in seconds using videotaped behavior software (Noldus Information Technology, EthoVision XT^®^).

### Biochemical Assessment of Serum Corticosterone Level

Following finishing behavioral tests, blood samples from the retroorbital venous plexus were obtained in EDTA-coated tubes from anesthetized mice, centrifuged for 10 min, and kept at −80°C for measuring the level of corticosterone by using radioimmunoassay (ELISA Kits; ALPCO Diagnostics, Orangeburg, NY).

#### Animal Dissection for Histological Study and Assessment of GFAP, Ki 67, and Caspase-3 Gene Expression Using Real-Time PCR

Immediately after taking the blood samples, the animals were sacrificed and the whole brain was carefully dissected with intact olfactory pulp, immersed in dried ice for farther dissection into left and right hemispheres, fixation in 10% neutral buffered formalin, and routinely processed for paraffin blocking in histopathology lab.

Paraffin processed samples were subjected to RNA extraction accord to methods adopted by from 100 mg of formalin-fixed paraffin-embedded (FFPE) sections obtained from the left brain hemisphere. They were deparaffinized in 1 ml of xylene, incubated at 56°C for 15 min, and centrifuged for 10 min at 13,000 g. The supernatant was discarded and the pellet washed twice with 1 ml 100% ethanol, centrifuged, the supernatant was discarded, and 1 ml Trizol was added to the pellet (25).

Extraction of total RNA using Trizol was done according to the supplier instruction (Invitrogen Life Technologies, Carlsbad, CA, USA). NanoDrop 2000 Spectrophotometer (Thermo Scientific, USA) was used to measure the concentration of RNA. Reverse transcription was done using oligo-dT primers (Bioneer Inc., Daejeon, Republic of Korea) in a 20-ll reaction including 5 ll RNA. The resulted complementary DNAs (cDNAs) were amplified using PCR Master Mix (Bioneer Inc., Daejeon, Republic of Korea) with primers (Metabion International AG, Semmelweisstr, Germany). GFAP gene (forward 5′-CAAGCCAGACCTCACAGCG-3′, reverse5′-GGTGTCCAGGCTGG-TTTCTC-3′, caspase-3 (forward 5′- *TGTATGCTTACTCTACCGCACCCG*-3′, reverse5′-*GCGCAAAGTGACTGGATGAACC*-3′), Ki67 (forward 5′-AAGAAGAGCCCACAGCACAGAGAA-3′, reverse5′-AAGAAGAGCCCACAGCACAGAGAA 3′), and β-actin (forward 5′-TCTGGCACCACA CCTTCTA-3′; reverse 5′-GGCATACAGGGACAGCAC-3′). PCR amplification was applied in a thermocycler (manufactured by Labnet International Inc.). The procedure was reported in a previous work of Ayuob, Firgany (24). Using comparative Ct method, normalization of current results to β-actin as a reference gene was done. Ct values were used to estimate the gene/β-actin ratio, with a value of 1.0 used as the control (calibrator). The normalized expression ratio was calculated using the 2−ΔΔCt (26). The mRNA level was expressed as a ratio or percent to that of corresponding β-actin

### Histopathological Assessment

Five microns serial paraffin sections from brain hemispheres including olfactory lobe were, stained with hematoxylin and eosin (H & E) for general histological assessment (27). Immunohistochemical staining was performed using the peroxidase-labeled streptavidin-biotin technique (28). Anti-glial fibrillary acidic protein (GFAP) antibody (DakoCytomation, Minneapolis, MN) was used for demonstration of astrocytes and was diluted 1:1,000 with phosphate-buffered saline (PBS). Anti-caspase-3 antibody (Santa Cruz Biotechnology, Santa Cruz, CA) was used to demonstrate apoptosis, and diluted to 1:1,000 with PBS. Assessment of indirect neurogenesis was assessed using anti-Ki-67 antibody (rabbit polyclonal IgG; Abcam, Cambridge, UK). It was diluted to 1:100 with PBS. To verify the specificity of each primary antibody, a negative control section was done by omitting the primary antibody. Examination of slides was performed using light microscope connected to a digital camera (Olympus, BX-61, Los Angeles, CA) for photographing.

The percentage area of GFAP immune-expression, caspase-3-positive cells and Ki67-positive cells was counted in five non-overlapping high power fields (x400) of MOB in each mouse [Makhlouf, El-Beshbishy (28)] using ImageJ 1.52a (National Institutes of Health, USA). The thickness of the mitral cell layer of the MOB was measured in five non-overlapping high power fields (x 400) in each animal.

### Statistical Analysis

Analysis of the data was carried out using the Statistical Package for the Social Sciences (SPSS, version 22) software. One-way ANOVA followed by LSD (least significant difference) *post hoc* were used to compare the parametric data of different groups. P values < 0.05 were considered significant.

## Results

### Behavioral Results

**Figure 1:**
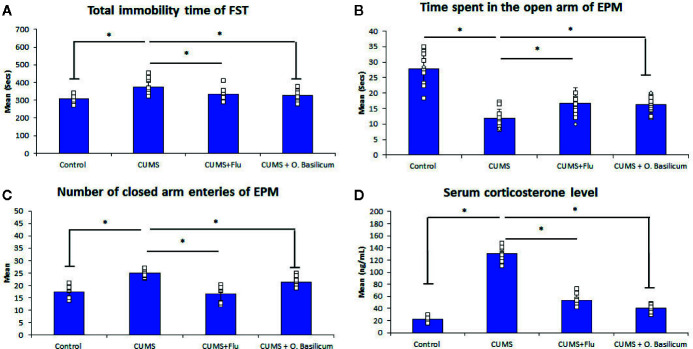
Effect of *Ocimum basilicum* on the immobility time of the forced swimming test (FST) **(A)**, the time spent in the open arm **(B)**, the number of closed arm entries **(C)** of the elevated plus maise (EPM) test, and serum corticosterone level **(D)**. Data are expressed as mean ± SD (n = 10). * significance with p < 0.05. CUMS, chronic unpredictable mild stress; FLU fluoxetine; *O. basilicum*, *Ocimum basilicum*.

Exposure of mice to CUMS for 4 weeks was found to result in significant increase in immobility time (p < 0.001). Administration of FLU or *O. basilicum* for 2 weeks after induction of depression significantly decreased immobility time compared to untreated mice (p = 0.02, p < 0.001) respectively (**Figure 1**).

CUMS also exhibited significantly decreased (p < 0.001) time spent in the open arms with a significant increase time spent (p < 0.001) in the closed arm during the EPM compared to control mice. On the other hand, both FLU and *O. basilicum* could significantly increase (p=0.001, p < 0.001) the duration the mice spent in the open arm during the EPM and significantly reduce (p < 0.001) entries to closed arms compared to untreated animals, respectively. This indicating the ability of FLU and *O. basilicum* to reduce the anxiety-like behavior in CUMS-exposed mice (**Figure 1**).

### Biochemical Results

#### Serum Corticosterone Level

Exposure of mice to CUMS for 4 weeks produced a significant increase (p < 0.001) in corticosterone serum level compared to the control. A significant reduction in corticosterone serum level was observed following treatment with either FLU (p < 0.001) or *O. basilicum* (p < 0.001) in comparison with untreated group (**Figure 1**).

#### Gene Expression of GFAP, Caspase-3, Ki 67

**Figure 2:**
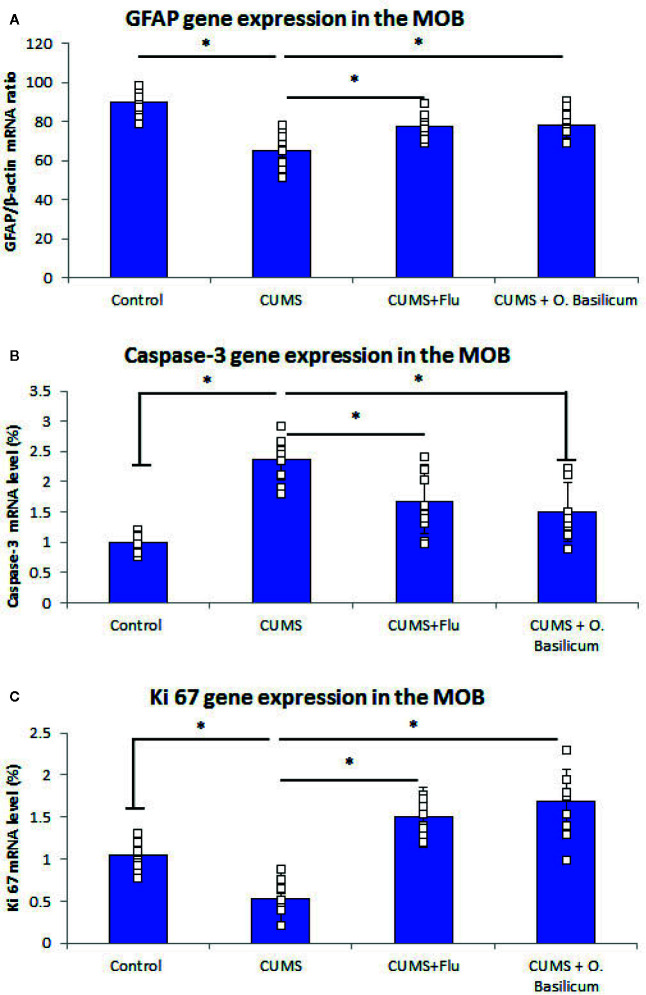
Effect of *Ocimum basilicum* on GFAP **(A)**, caspase-3 **(B)**, Ki 67 **(C)** mRNA levels detected by real-time PCR (RT-PCR) in the main olfactory bulb (MOB) after treatment of chronic unpredictable mild stress (CUMS) in rats with fluoxetine (FLU) and *O. basilicum*. The messenger RNA (mRNA) level was expressed as a ratio or percent to that of corresponding β-actin. Data were presented as mean ± SD (n=10 in each group). * significance with p<0.05.

Real-time PCR (RT-PCR) revealed significantly lowered (p < 0.001) GFAP gene expression in MOB of CUMS group in comparison with control while FLU- or *O. basilicum*-treated groups showed significant higher levels (p = 0.003, p<0.001) in comparison with untreated CUMS exposed mice, respectively (**Figure 2**).

On the other hand, caspase-3 gene expression was significantly increased (p < 0.001) in the MOB of CUMS group, while it was reduced significantly (p = 0.002, p<0.001) in both FLU- or *O. basilicum*-treated groups compared to untreated CUMS group, respectively (**Figure 2**).

Ki67 gene expression, was reduced significantly (p < 0.001) in MOB of CUMS group, in comparison to significant higher level in the groups received FLU- or *O. basilicum* (p = 0.002, p < 0.001) in comparison with untreated CUMS group respectively (**Figure 2**).

### Histopathological Results

Examination of MOB of control group (H&E stain) showed that it was consisted of six distinct layers include from outside inward; olfactory nerve of multipolar nerve cells called mitral cells, glomerular, external plexiform, mitral cell, internal plexiform, with the most inner granular cell layer. The mitral cells have large vesicular lightly stained nuclei with well-defined nucleoli and abundant amount of basophilic granular cytoplasm. Smaller nerve cells from granular cell layer, with dark nuclei and little amount of cytoplasm, appeared scattered between the mitral cells.

**Figure 3:**
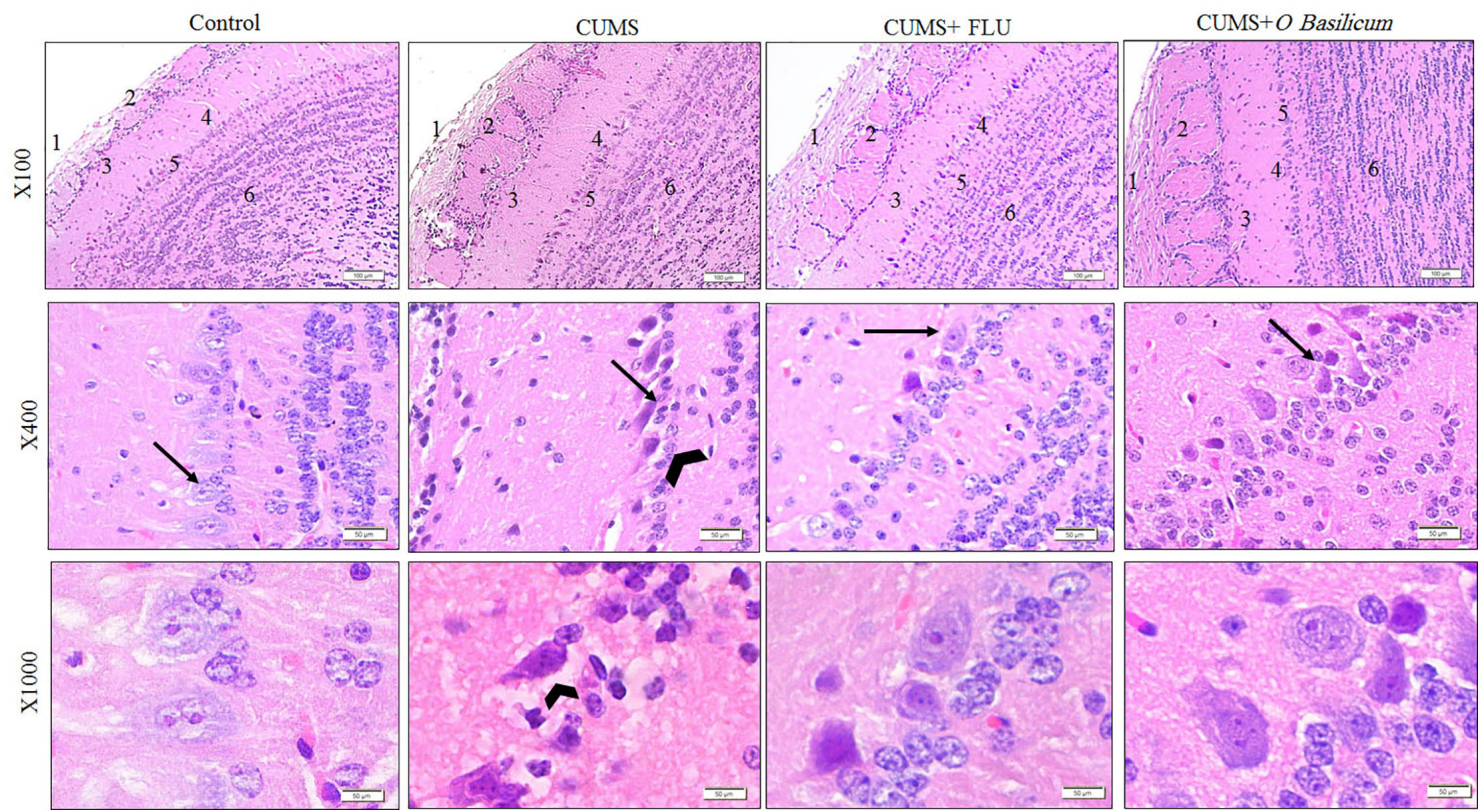
The histological structure of the main olfactory bulb (MOB) of the studied groups show nerve fiber layer (1), glomerular layer (2), external plexiform layer (3), mitral layer (4), internal plexiform layer (5), and granular layers (6). Note the affected mitral layer in the CUMS group appear with higher magnification and show distorted and small Mitral cells (arrow) compared with other group together with widening of pericellular space around them (arrow head). H&E staining. CUMS, chronic unpredictable mild stress; FLU, fluoxetine; *O. basilicum*, *Ocimum basilicum*.

**Figure 4:**
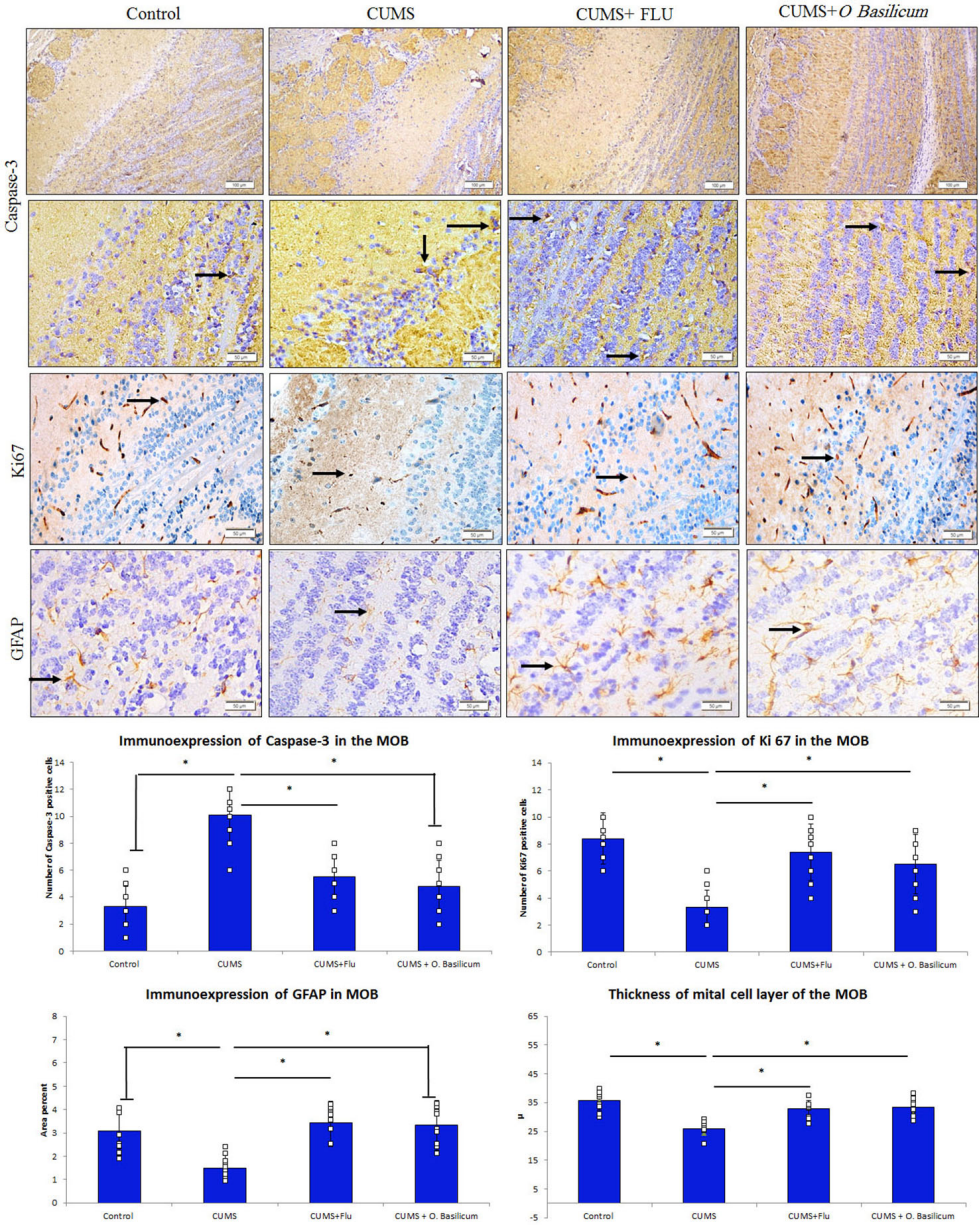
Effect of *Ocimum basilicum* on the immunoexpression of caspase-3, Ki67, and GFAP in the MOB of the studied groups. Main olfactory bulb (MOB), chronic unpredictable mild stress (CUMS), fluoxetine (FLU), *O. basilicum (Ocimum basilicum).* * significance with p < 0.05.

Examination of the MOB of the CUMS group revealed that the mitral cell layer was affected where nerve cells, in this layer, appeared smaller and distorted in shape and had deeply stained cytoplasm, dark pyknotic nuclei, and wide pericellular spaces (**Figure 3**). The thickness of this layer was significantly reduced in this group (p < 0.001) in comparison to control. Groups treated with FLU or *O. basilicum* showed restoration of the normal appearance of mitral nerve cells while few mitral cells appeared distorted with significant increase in thickness (p < 0.001) when compared to untreated CUMS mice. Insignificant difference could be recognized between the two animal groups (p=0.035) (**Figures 3** and **4**).

### Immunoexpression of Caspase-3, Ki 67, and GFAP in the Main Olfactory Bulb

It was observed that caspase-3 immunoexpression was increased in the MOB of CUMS group, while it was reduced back in both FLU- and *O. basilicum*-treated groups (**Figure 4**). A significant increase (p < 0.001) in caspase-3 positive cells in the MOB of CUMS group was observed upon statistical analysis of area density in comparison to control mice. In FLU- and *O. basilicum*-treated groups, expression was significantly lower (p < 0.001) compared to the untreated mice. There was insignificant difference (p = 0.39) in caspase-3 positive cells between FLU- and *O. basilicum*-treated groups (**Figure 4**).

Regarding GFAP immunoexpression, the astrocytes processes in various layers of the control MOB showed moderate positive immunoexpression. A significant reduction (p = 0.001) in percentage area of GFAP immune expression in the MOB of CUMS group was observed upon statistical analysis compared to control animals.

On contrast, a significant rise (p < 0.001) in GFAP immunoexpression was recorded in FLU- and O. *basilicum*-treated groups compared to the untreated mice. Insignificant difference (p = 0.81) was observed between FLU- and *O. basilicum*-treated groups (**Figure 4**).

Many nerve cells in the MOB exhibited immune positive expression for Ki 67. Statistical analysis revealed a significant (p < 0.001) reduction in Ki67-positive cells in the MOB in comparison to its expression in control mice while a significant increase was found in FLU- and O. *basilicum*-treated groups (p < 0.001) in comparison with untreated CUMS group with no significant difference (p = 0.30) between the two groups (**Figure 4**).

## Discussion

Olfactory function plays an important role in health and behavior. The laminar organization of olfactory bulb combines a unique neuronal morphology and complex synaptic connections which play a basic role in smell perception and modify the response of the output neurons for sharp tuning of a given odor (29). Olfactory dysfunction is considered a premotor sign of neurodegeneration that appears early in the degenerative process as in Alzheimer disease and Parkinson disease (30, 31). Moreover, the olfactory bulbectomy of rats has been validated as a model of depression over the past 30 years, suggesting a close relationship between MOB and depression (15–17). Reviewing literature showed that the, pathophysiology of MOB induced by CUMS is still unclear. Therefore, the present study was designed to describe the pathological alteration occurred in MOB of mice after exposure to CUMS and to investigate the efficacy of inhalation of *O. basilicum* essential oils in improving these changes as well as to understand the possible mechanism underlying this effect.

Mice exposed to the CUMS, in this study, showed depressive-like behavior verified by increased time of immobility during the FST and confirmed by decreasing the time spent in the open arms of the EPM as well as increasing of serum level of corticosterone. Similar results were reported by (32).

In this study, FLU induced an improvement in the behavioral, biochemical, and structural changes occurred in MOB of mice exposed to CUMS. In consistent with these findings, some previous studies revealed that FLU alleviated the behavioral changes induced by CUMS and improve the CUMS-induced structural changes in the hippocampus (33).

It was stated that stress enhanced lipid peroxidation and decreased oxidative stress defense in depressed patients that endorses undesirable effects on many cellular functions as showed by decreased plasma antioxidant defenses (34). It was reported that oxidative stress has a crucial role in the pathophysiology of depression in rodents and humans (35) and (36). Methanol, ethanol, or water extracts of *O. basilicum* seeds were described to have good antioxidants activity (9). Therefore, *O. basilicum* was used, in this study, to relieve the CUMS-induced changes on the MOB. Inhalation of *O. basilicum* alleviated this depressive status, evident by the behavioral tests, and reduced elevated corticosterone levels documented in animals subjected to CUMS. It was proposed that *O. basilicum* antidepressant capacity might be related to the increased brain level of enzymatic and non-enzymatic antioxidants endorsed by *O. basilicum* extracts (37).

This work demonstrated that exposure to CUMS caused structural alteration in the olfactory bulb of mice represented by distortion and shrinkage of mitral cells. This might be responsible for the atrophy reported in this study as well as some previous studies. Exposure to CUMS was reported to result in cortical and limbic brain regions atrophy that including also hippocampus and MOB (38) elevation of inflammatory mediators in hippocampal regions (39), and disturbances in hypothalamic-pituitary-adrenal (HPA) axis that might explain the increased corticosterone level in CUMS mice (40). *O. basilicum* was found to ameliorate CUMS-induced neuronal changes in MOB especially mitral cells. This effect might be attributed to the bioactive compounds present in *O. basilicum* essential oils like linalool, eugenol, cineole, and many other compounds that exert free radical scavenging activity (8, 24). These essential oils inhibit liposomal peroxidation and scavenge hydroxyl radicals, NO, and superoxide anion.

GFAP immunoreaction was used as a selective marker for estimation of astrocytes integrity in MOB as reported by (41). In the present study, administration of FLU and *O. basilicum* reversed the CUMS-induced reduction in GFAP expression. This was for a certain extent is in agreement with (32) who reported that fluoxetine could prevent GFAP reduction and glial atrophy and restore the integrity of astrocytes in CUMS-induced animal model of depression. *O. basilicum*, in this study, could be considered to have a similar effect as fluoxetine. In consistent with this study (14), recently reported that *O. basilicum* (at the dose of 100 and 200 μl/kg) induced neuroprotective effect against ethidium bromide-induced cognitive deficit through amelioration of neuroinflammation, mitochondrial dysfunction, and astrogliosis in the prefrontal cortex of the animals.

The olfactory bulb together with hippocampal dentate gyrus subventricular and the sub-granular zones, represent the three regions in the brain that undergo adult neurogenesis; the process that maintains continuous turnover of bulbar interneurons. Granular and periglomerular neurons of OB are well known to be differentiating and from cell precursors located in the subventricular zone and migrating to OB where it can respond to odor stimulations (42–44). In this study, Ki67 positive cells, which represent the newly proliferating nerve cells, was observed in the control MOB. This was previously described by (45). Exposure to CUMS resulted in a significant decrease in proliferating cells number in MOB of CUMS-exposed mice and this finding was consistent with that of Ki67 gene expression that revealed a considerable down-regulation in CUMS animals when compared to control. Similar results were recorded by (46) and (47) in the MOB of chronic stressed rats using the cell proliferation markers; PSA-NCAM, DCX, and BrdU. In addition, previous studies reported a significant suppression of neurogenesis in hippocampal regions in mice model of depression (12, 48). This effect might be attributed to increased plasma levels of corticosterone in CUMS-exposed rats, recorded in this study as well as many other studies which concluded that chronic exposure to corticosterone, or stressors that increase its secretion, has powerful suppressor effect on proliferation process (10, 49, 50).

Administration of FLU and *O. basilicum* significantly improved the neurogenesis and evidenced by upregulated Ki67 gene expression. This was supported by other studies conducted on MOB of mice (47), hippocampus of mice (51), and hippocampus of human (52). Brain derived neurotropic factor and other neurotrophins expressions were reported to be increased by antidepressant drugs and thus stimulate neurogenesis and repairing of damaged neurons (24, 53).

In this study, increased neuronal apoptosis, evidenced by increase number of caspase-3 positive cells as well as upregulation of caspase-3 gene expression was detected in MOB of mice exposed to CUMS. In agreement with this (54), and Meyer, Glaser (4) reported marked increase of apoptosis in rat and mice olfactory bulbs in models of experimentally induced neurodegeneration as well as in the hippocampus of CUMS-exposed mice (48).

In the present study, *O. basilicum* reduced caspase-3 positive cells population *via* downregulated caspase-3 gene expression. In consistent with such finding neuroprotective effect of O. *basilicum* with reduced size of cerebral infarct and lipid peroxidation were described in the brain by (55). It reduced the corticosterone level, apoptosis of hippocampal neurons, and increased both newly formed nerve cells and astrocytes numbers in a manner comparable to FLU (10). This effect was attributed to effect of phenolic, flavonoids, and tannin contents of *O. basilicum* essential oils which were previously reported to act as reactive oxygen species scavengers [Garabadu and Singh (14)] recently described that *O. basilicum* attenuated significantly mitochondria-dependent apoptosis induced by ethidium bromide in rat prefrontal cortex.

Among the limitations of this study was the absence of the in-depth analysis of the detailed mechanism of the *O. basilicum* neuroprotective effect which needs further future study.

## Conclusion

The present paper showed that *O. basilicum* relieved depressive-like behavioral alterations induced in mice following exposure to chronic mild stress. O. *basilicum* also was found to ameliorate stress-induced changes in the main olfactory bulb as evident both biochemically and histopathologically. These effects might be mediated down-regulation of caspase-3 in addition to up-regulation of GFAP and Ki67 gene expression in the MOB.

## Data Availability Statement

The original contributions presented in the study are publicly available. This data can be found here: https://figshare.com/s/d5a87f5869ea80e2598f.

## Ethics Statement

The animal study was reviewed and approved by Biomedical research ethics committee, Faculty of medicine, Kind Abdulaziz University, Jeddah, Saudi Arabia.

## Author Contributions

NA and SA designed the study, conducted the analyses and wrote the initial version of this manuscript. MB, IA, HA, and AA collected the samples, data, performed the literature review and interpret the results. All authors contributed to the article and approved the submitted version.

## Conflict of Interest

The authors declare that the research was conducted in the absence of any commercial or financial relationships that could be construed as a potential conflict of interest.
