# Corrigendum: *Ocimum basilicum* (Basil) Modulates Apoptosis and Neurogenesis in Olfactory Pulp of Mice Exposed to Chronic Unpredictable Mild Stress

**DOI:** 10.3389/fpsyt.2021.781922

**Published:** 2021-11-03

**Authors:** Nasra N. Ayuob, Maha J. Balgoon, Soad Ali, Ibrahim S. Alnoury, Hailah M. ALmohaimeed, Amany A. AbdElfattah

**Affiliations:** ^1^Department of Medical Histology and Cell Biology, Faculty of Medicine, Damietta University, Damietta, Egypt; ^2^Department of Medical Histology, Faculty of Medicine, Delta University for Science and Technology, Mansoura, Egypt; ^3^Yousef Abdullatif Jameel, Chair of Prophetic Medical Applications (YAJCPMA), Faculty of Medicine, King Abdulaziz University, Jeddah, Saudi Arabia; ^4^Department of Biochemistry, Faculty of Science, King Abdulaziz University, Jeddah, Saudi Arabia; ^5^Department of Anatomy, Faculty of Medicine, King Abdulaziz University, Jeddah, Saudi Arabia; ^6^Department of Histology, Faculty of Medicine, Assuit University, Assuit, Egypt; ^7^Department of ENT, H&N Surgery, Faculty of Medicine, King Abdulaziz University Hospital, Jeddah, Saudi Arabia; ^8^Department of Basic Science, Medical College, Princess Noruh Bint Abdulrahman University (PNU), Riyadh, Saudi Arabia; ^9^Department of Histology and Cell Biology, Faculty of Medicine, Mansoura University, Mansoura, Egypt

**Keywords:** *Ocimum basilicum*, chronic stress, caspase-3, anti-glial fibrillary acidic protein, olfactory bulb, neurogenesis

In the published article, there was an error in affiliation 6. Instead of “Abdul Aziz,” it should read “Abdulaziz.”

In the published article, there was an error regarding the affiliation(s) for Nasra N. Ayuob. As well as having affiliations 2 and 3, they should also have *Department of Medical Histology and Cell Biology, Faculty of Medicine, Damietta University, Damietta, Egypt*.

Therefore, affiliation 6 has now been renumbered affiliation 7.

The authors apologize for these errors and state that this does not change the scientific conclusions of the article in any way. The original article has been updated.

## Publisher's Note

All claims expressed in this article are solely those of the authors and do not necessarily represent those of their affiliated organizations, or those of the publisher, the editors and the reviewers. Any product that may be evaluated in this article, or claim that may be made by its manufacturer, is not guaranteed or endorsed by the publisher.

